# Development of a Gold Nanoparticle-Based Immunochromatographic Strip for Rapid Detection of Porcine Circovirus Type 2

**DOI:** 10.1128/spectrum.01953-22

**Published:** 2023-07-19

**Authors:** Min Jiang, Aiping Wang, Yaning Sun, Yuan Li, Yumei Chen, Jingming Zhou, Hongliang Liu, Peiyang Ding, Yanhua Qi, Ning Li, Gaiping Zhang

**Affiliations:** a Longhu Laboratory of Advanced Immunology, Zhengzhou, Henan, China; b School of Life Sciences, Zhengzhou University, Zhengzhou, Henan, China; c School of Advanced Agricultural Sciences, Peking University, Beijing, China; d Henan Agricultural University, Zhengzhou, Henan, China; e Henan Provincial Key Laboratory of Immunobiology, Zhengzhou, China; f School of Life Sciences, Lanzhou University, Lanzhou, Gansu, China; University of Prince Edward Island

**Keywords:** porcine circovirus type 2, monoclonal antibody, rapid detection, immunochromatographic strip, capsid protein

## Abstract

Porcine circovirus type 2 (PCV2) is an important swine infectious pathogen that seriously threatens the global swine industry. PCV2 Cap protein is the only structural and the main immunogenic protein constituting the viral capsid. In this study, a gold nanoparticle-based immunochromatographic strip with high sensitivity and specificity was developed which could be used for rapid detection of PCV2 virions or Cap protein in research. The visual detection limit of the strip was 10^3.18^ 50% tissue culture infective does (TCID_50_)/mL for PCV2, and 2.03 μg/mL for PCV2 Cap protein. No cross-reactivity was observed with the PCV1 and PCV3 Cap proteins and other common swine pathogens such as porcine reproductive and respiratory syndrome virus, classical swine fever virus, pseudorabies virus, porcine epidemic diarrhea virus, porcine parvovirus, and swine influenza virus. The repeatability of the strip was good. The stability of the strip was perfect for 12 months in a dry state at room temperature. Visual results could be obtained within 5 min by simply inserting the strip into the diluted sample. The strip is a time-saving, labor-saving, and reliable tool for testing of PCV2 virions or Cap protein in research. The idea of this study might open a new perspective for the application of the strip.

**IMPORTANCE** Porcine circovirus type 2 (PCV2) Cap protein is the only structural and the main immunogenic protein constituting the viral capsid. Although many methods can be used to identify PCV2 or PCV2 Cap protein in vaccine research, they usually require high workload and time. The developed strip can specifically detect PCV2 virions or Cap protein, and visual qualitative results can be obtained within 5 min by simply diluting the sample and inserting the strip into the sample. The final value of the strip is providing a simple and time-saving method for real-time monitoring of PCV2 antigen in vaccine research with reliable results, such as the different stages of PCV2 Cap protein expression and purification, as well as the different stages of PCV2 reproduction and purification.

## INTRODUCTION

Porcine circovirus (PCV) belongs to the family *Circoviridae* and the genus *Circovirus* and is a small, nonenveloped, single-stranded DNA virus (1, 2). Currently, there are four genotypes of PCV, PCV1, PCV2, PCV3, and PCV4. PCV1, considered nonpathogenic to pigs, was originally separated from the porcine kindey-15 (PK-15) cell line as a contaminant of cell culture (3). In contrast, PCV2 is the primary causative agent of porcine circovirus-associated diseases (PCVAD). The economic impact of PCVAD is tremendous, especially as postweaning multisystemic wasting syndrome (4, 5). PCV3, with a circular genome of 2,000 nucleotides distantly related to known circoviruses, is a novel porcine circovirus, which may play an etiologic role in reproductive failure and porcine dermatitis and nephropathy syndrome (6–9). PCV4 is a novel porcine circovirus discovered by high-throughput sequencing, and its pathogenic mechanism is still unclear (10). PCV2 remains the main infectious pathogen of PCVAD and the focus of prevention and control in the global swine industry.

PCV2 infection not only causes PCVAD, but also suppresses the immune system of pigs, thus increasing the probability of infection with other pathogens such as porcine reproductive and respiratory syndrome virus (PRRSV), porcine parvovirus (PPV), and Mycoplasma pneumoniae, aggravating the severity of the disease and causing serious economic losses. Vaccination is a powerful tool to control PCVAD, and antigen is essential to ensure the effectiveness of PCV2 vaccine. At present, PCV2 vaccines include inactivated virus vaccines, chimeric vaccines, and subunit vaccines (11–13). Effective antigens of PCV2 vaccines are mainly PCV2 Cap protein, PCV2 inactivated virus, or chimeric virus with PCV2 Cap protein. There are various methods to detect PCV2 antigen in research, such as Western blotting (WB), indirect immunofluorescence assay (IFA), and enzyme-linked immunosorbent assay (ELISA) (14–18). However, these methods are time-consuming, laborious, and expensive.

In this study, a gold nanoparticle (AuNP immunochromatographic strip based on highly sensitive and specific anti-PCV2 monoclonal antibodies [MAbs]) was developed, providing a one-step, time-saving, labor-saving method for detecting PCV2 and Cap protein in vaccine research, such as the different stages of virus production and protein expression, with reliable visual results in 5 min. The idea of this study might open a new perspective for the application of the strip.

## RESULTS

### Preparation and characterization of the monoclonal antibodies.

**FIG 1:**
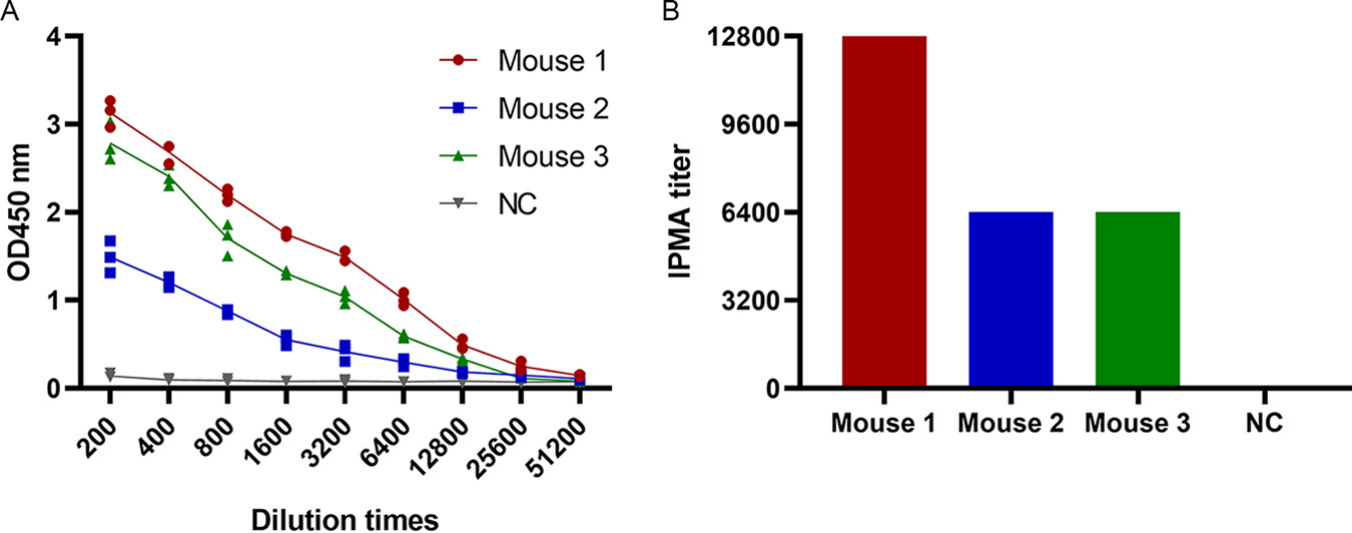
Serum titers of immunized mice at 42 dpi. (A) Titers of serum samples were detected by ELISA. (B) Titers of serum samples were detected by IPMA. Negative control (NC), serum sample from the mouse mock-immunized with phosphate-buffered saline (PBS). OD_450_, optical density at 450 nm.

**FIG 2:**
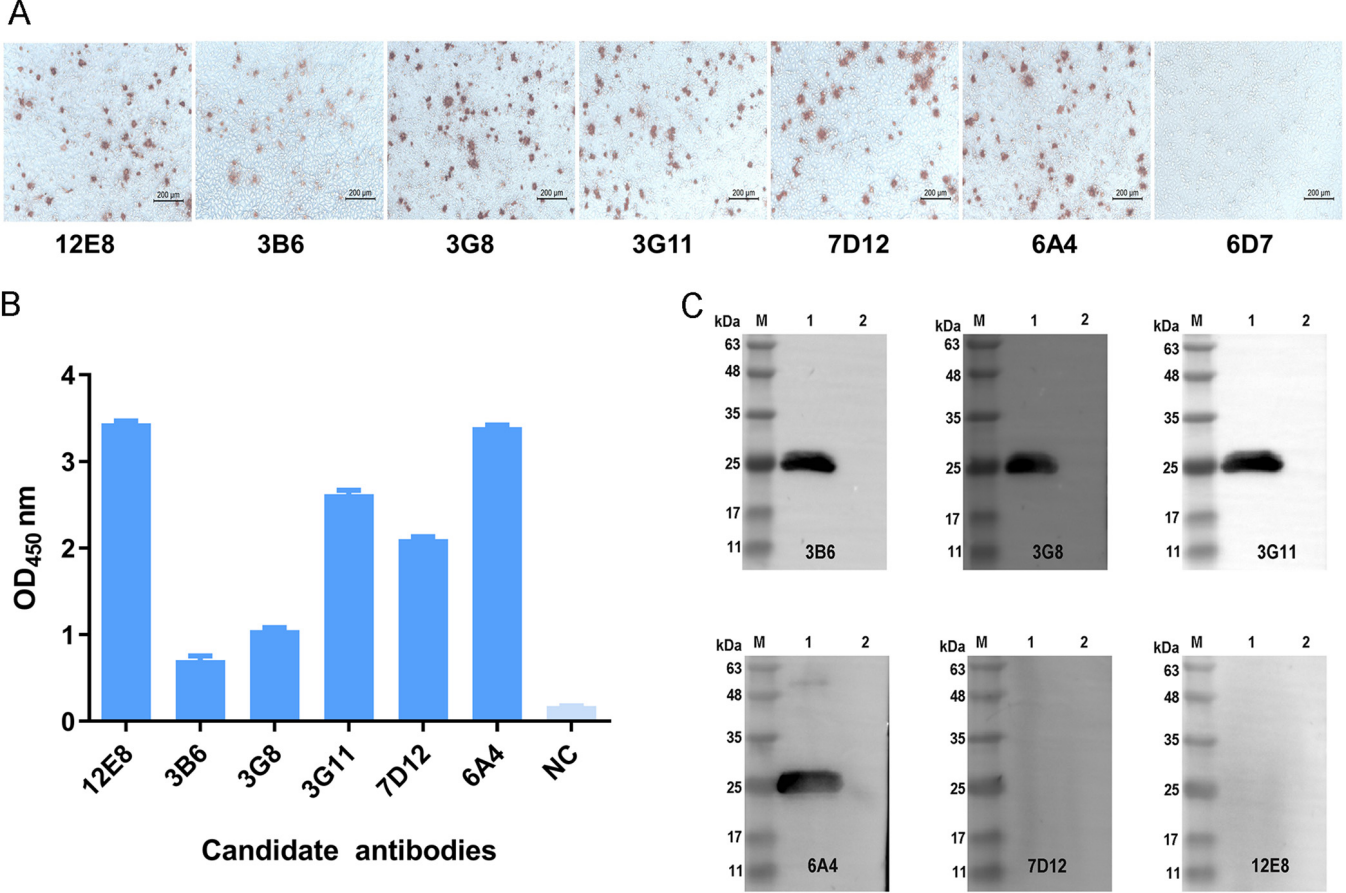
Characterization of the MAbs. (A) Reactivity of the MAbs with the PCV2 strain HN-LB-2016. 6D7, a MAb against PCV3 used as negative control. (B) Reactivity of the MAbs with PCV2 Cap protein. NC, MAb against PCV3 was used as a negative control. (C) Reactivity of the MAbs with the denatured Cap protein. M, marker; lane 1, PCV2 Cap protein; lane 2, PET28a empty vector. OD_450_, optical density at 450 nm.

The results from ELISA and immune peroxidase monolayer assay (IPMA) showed that Mouse 1 had the highest titers and was selected as the spleen donor to generate MAbs against PCV2 (Fig. 1). Six monoclonal antibodies against PCV2 were screened by IPMA and were named 3B6, 3G8, 3G11, 6A4, 7D12, and 12E8 (Fig. 2A). Results from ELISA showed that all 6 MAbs effectively recognized PCV2 Cap protein, while 12E8, 3G11, and 6A4 reacted more actively (Fig. 2B). Results from WB showed that 3B6, 3G8, 3G11, and 6A4 reacted with denatured PCV2 Cap protein, but 7D12 and 12E8 did not, indicating that 3B6, 3G8, 3G11, and 6A4 recognized linear epitopes on PCV2 Cap protein, while 7D12 and 12E8 might recognize a conformational epitope (Fig. 2C). Results from isotype identification showed that the heavy chains of three MAbs were IgG2a, those of two were IgG2b, that of one was IgG3, and the light chains of all MAbs was κ (Table 1). The IPMA titers of the cell culture supernatant and the ascites titers of these MAbs ranged from 320 to 1,280 and 32,000 to 256,000, respectively, and the antibody strip titers were 160 to 640 and 16,000 to 128,000, respectively (Table 1). There was no cross-reaction between the MAbs and other swine viruses, including PRRSV, classical swine fever virus (CSFV), pseudorabies virus (PRV), porcine epidemic diarrhea virus (PEDV), and PPV, indicating that these MAbs had high specificity (Table 1).

**TABLE 1:** Characteristics of MAbs in this study

MAb	Subtype	IPMA titer (PCV2)		Strip titer (PCV2 Cap protein)		VN titer (PCV2)^*a*^		Cross-reactivity^*b*^				
		Supernatants	Ascites	Supernatants	Ascites	Supernatants	Ascites	PRRSV	CSFV	PRV	PEDV	PPV
3B6	IgG2a, κ	1,280	25,6000	160	16,000	32	512	–	–	–	–	–
3G8	IgG2a, κ	640	64,000	320	64,000	8	128	–	–	–	–	–
3G11	IgG2a, κ	640	64,000	160	16,000	NC	/	–	–	–	–	–
6A4	IgG2b, κ	1280	128,000	640	128,000	NC	/	–	–	–	–	–
7D12	IgG3, κ	320	32,000	320	64,000	128	2,048	–	–	–	–	–
12E8	IgG2b, κ	640	128,000	640	128,000	NC	/	–	–	–	–	–

a NC, mAbs had no neutralizing activity; /, not tested.

b –, MAbs did not cross-react with other swine viruses.

### Selection of the matched antibody pair.

**FIG 3:**
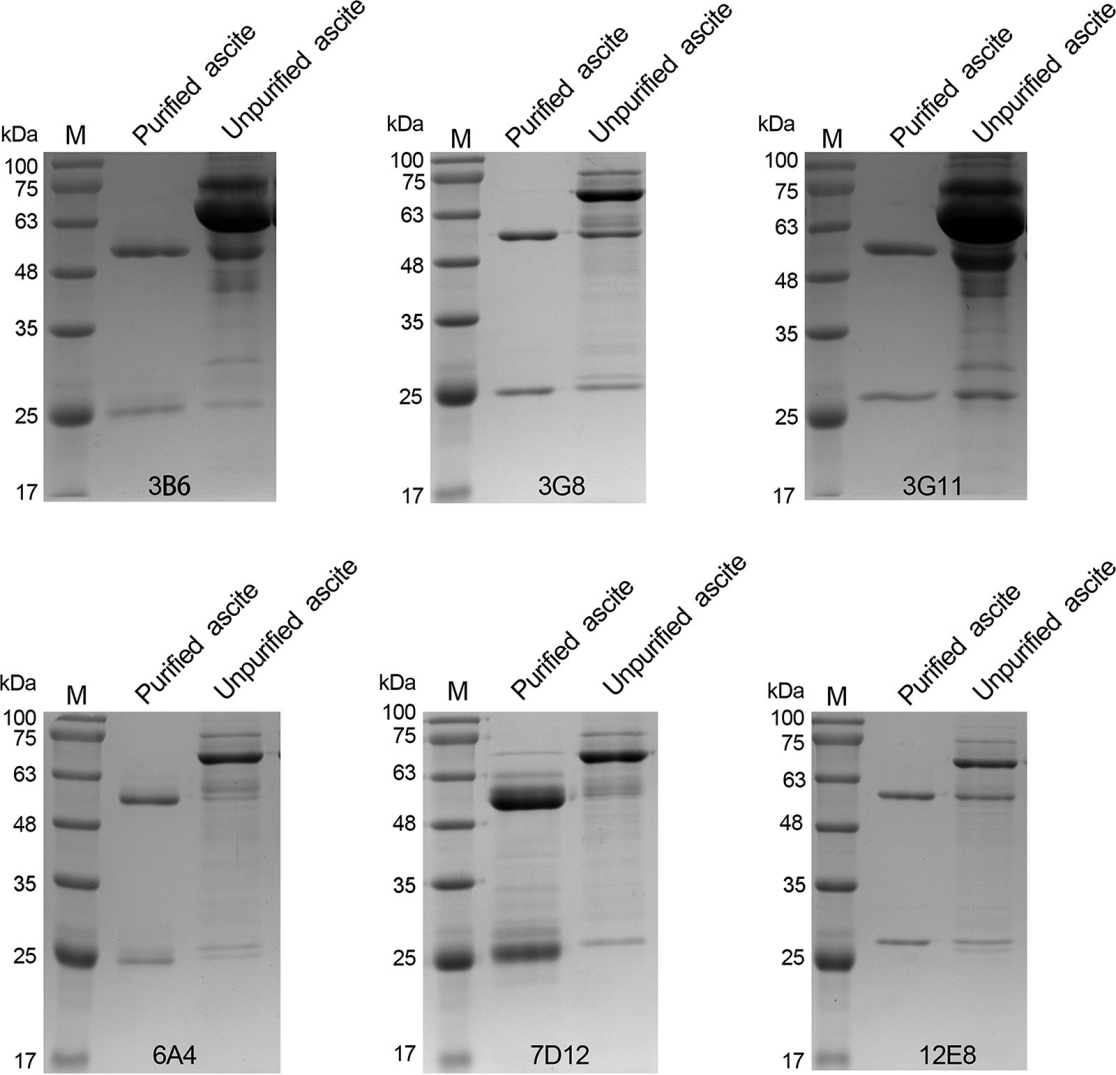
Purification of the ascites containing the MAbs. Before being labeled with AuNPs, the MAbs were purified by protein G affinity chromatography. Lane 1, the purified MAb ascites; lane 2, the unpurified MAb ascites.

**FIG 4:**
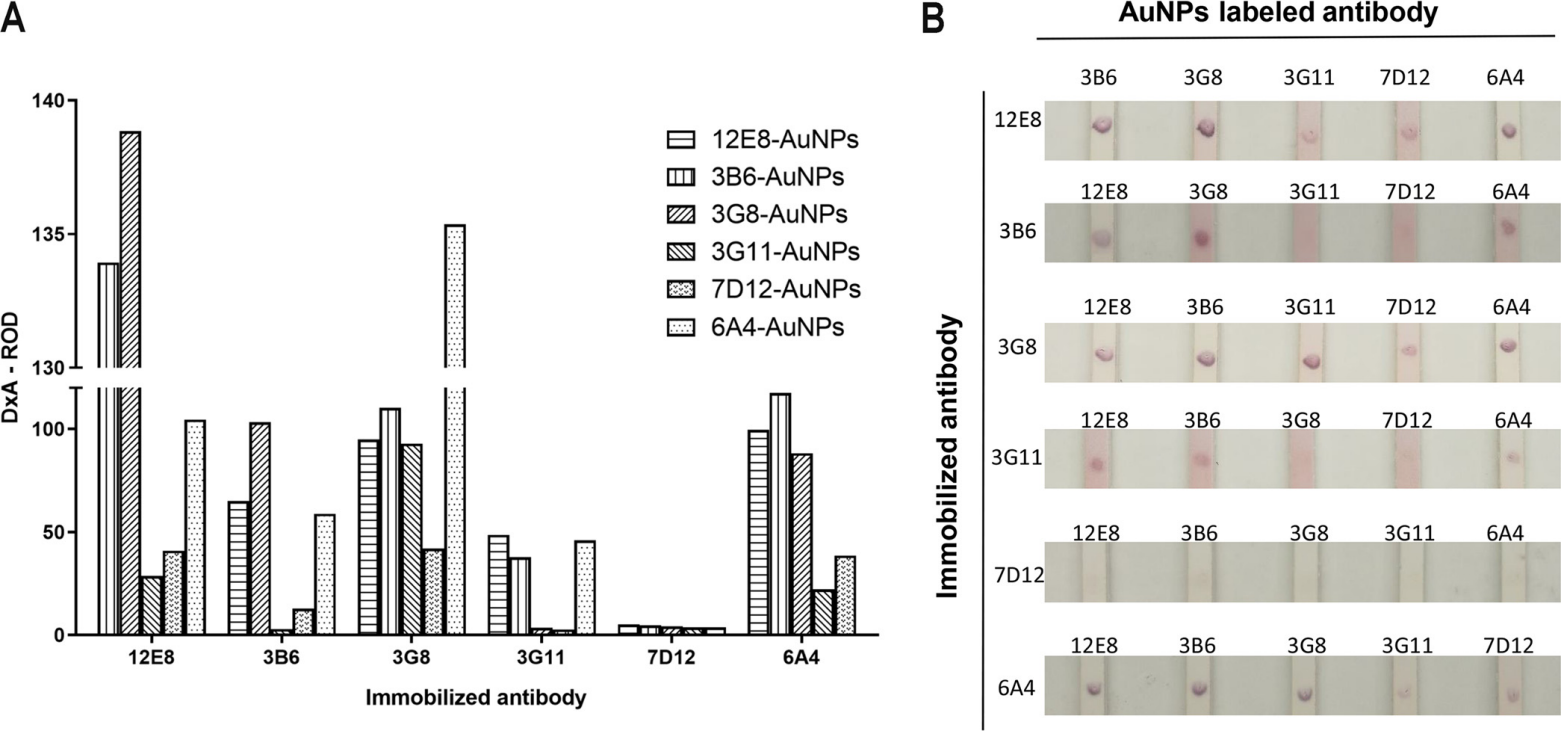
Selection of the matched antibody pair. (A) Color intensity of the dots. DxA-ROD, relative optical density of the whole screened area values. (B) The 2-fold serially diluted PCV2 strain HN-LB-2016 (1.95 × 10^5^ TCID_50_/mL) was used to detect the dot strips.

Under reducing conditions, the heavy chain of IgG antibody is approximately 50 kDa and the light chain is approximately 25 kDa. SDS-PAGE results showed that bands were observed at about 50 kDa and 25 kDa, and no other obvious band was observed, indicating that the purified MAbs had high purity (Fig. 3). As shown in Fig. 4, the matched antibody pair (MAb 12E8 and 3G8-AuNPs) displayed the strongest reactivity to PCV2. The matched antibody pairs (12E8 and 3B6-AuNPs, 3G8 and 6A4-AuNPs) showed similar reactivity with the matched antibody pair (MAb 12E8 and 3G8-AuNPs). Based on the above-described results, these three matched antibody pairs had potential to be selected to prepare the strip for the rapid detection of PCV2 and PCV2 Cap protein. For ease of operation, we selected antibody pair 12E8 and 3G8-AuNPs for subsequent experiments.

### Characterization of MAb-AuNPs.

**FIG 5:**
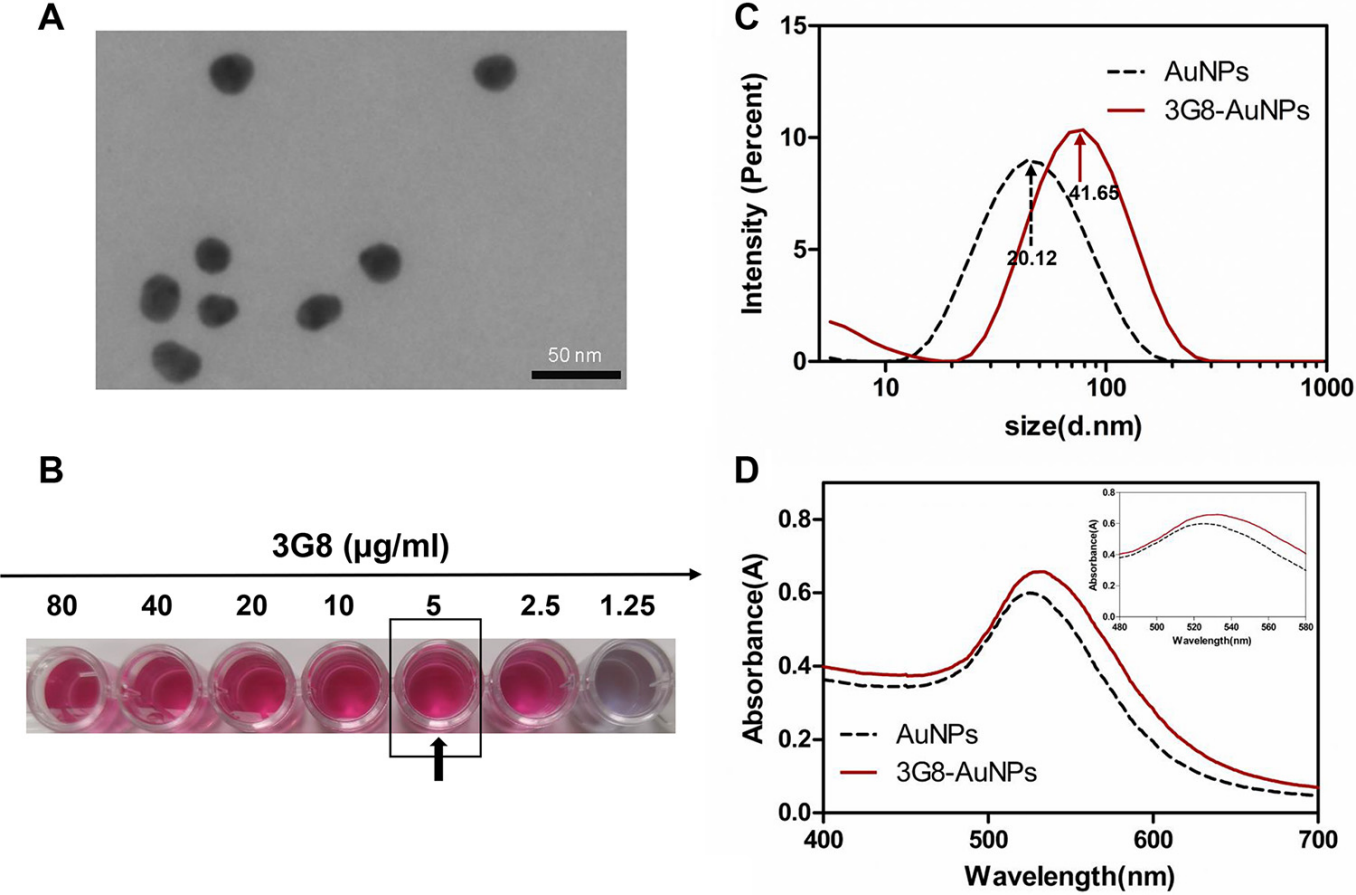
Characterization of 3G8-AuNPs. (A) Transmission electron micrograph (TEM) of AuNPs. Scale bars = 50 nm. (B) The optimal concentration of 3G8 for conjugation. The arrow indicates the optimal concentration. (C) Dynamic light scattering (DLS) of AuNPs and 3G8-AuNPs. (D) UV-vis absorption spectra of AuNPs (λ_max_ = 525 nm) and 3G8-AuNPs (λ_max_ = 532 nm).

The transmission electron microscopic (TEM) image showed that AuNPs were synthesized and had a well-dispersed distribution (Fig. 5A). When 10% NaCl was added into the AuNP solution, the lowest concentration of MAb 3G8 required to stabilize the AuNPs was 5 μg/mL (Fig. 5B). Before being conjugated with MAb 3G8, the average diameter of AuNPs was 20.12 nm and the absorption peak was 525 nm. After MAb was conjugated to AuNPs, the average diameter increased to 41.65 nm, and the maximum absorbance shifted to 532 nm (Fig. 5C and D). All results indicated that 3G8-AuNPs were well prepared, laying the foundation for the preparation of the strip for the rapid detection of PCV2 and PCV2 Cap protein.

### Sensitivity of the immunochromatographic strip.

**FIG 6:**
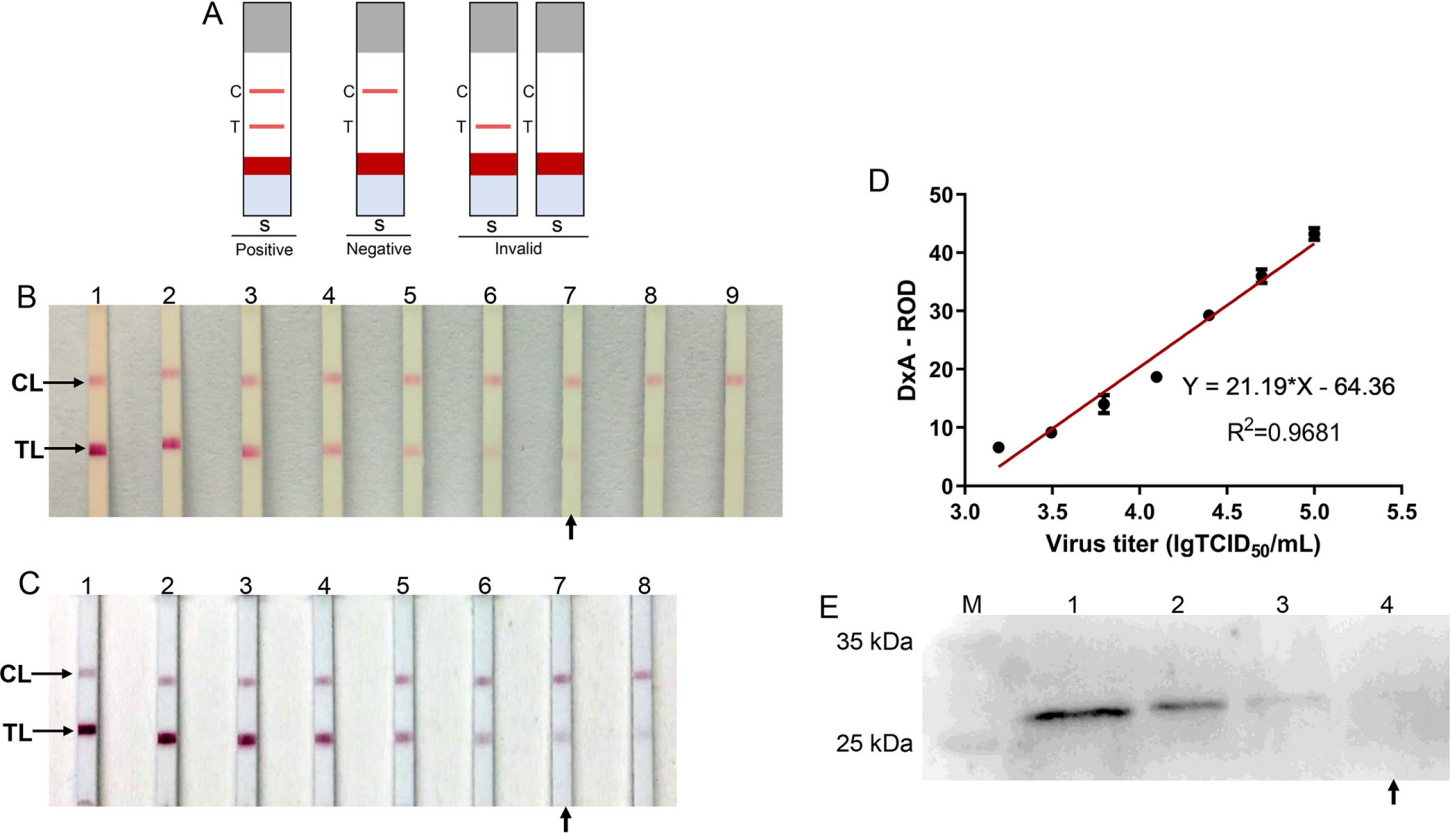
Sensitivity of the immunochromatographic strip. (A) Schematic diagram of the result judgment. (B) Sensitivity of the strip for PCV2. Lanes 1 to 9, 2-fold serially diluted PCV2 ranging from 10^5.00^ to 10^2.58^ TCID_50_. (C) Sensitivity of the strip for PCV2 Cap protein. Lanes 1 to 8, 2-fold serially diluted PCV2 Cap protein ranging from 130 to 1.02 μg/mL. CL, control line. TL, test line. The arrow indicates the LOD. (D) Correlation analysis between DxA-ROD values of the strip and virus titer. (E) Detection of PCV2 Cap protein by Western blotting. Lanes 1 to 4, 2-fold serially diluted PCV2 Cap protein ranging from 130 to 16.25 μg/mL.

The 2-fold serial dilutions of PCV2 strain HN-LB-2016 ranging from 10^5.00^ to 10^2.58^ TCID_50_ and PCV2 Cap protein ranging from 130 to 1.02 μg/mL were used to determine the limit of detection (LOD) of the strip. The LOD of the strip to detect PCV2 strain HN-LB-2016 was 10^3.18^ TCID_50_ (Fig. 6B), and the LOD of the strip to detect PCV2 Cap protein was 2.03 μg/mL (Fig. 6C). As shown in Fig. 6D, there was a linear relationship between the relative optical density of the whole screened area (DxA-ROD) values of the strip and virus titers, and the correlation coefficient was 0.9681, indicating that the color shades on the test line (TL) reflected the antigen content to a certain extent. WB is the gold standard for the detection of protein antigens in laboratory research. The sensitivity of the strip was compared to that of WB. It shows that the visual LOD of WB to detect PCV2 Cap protein was 16.25 μg/mL. The strip for detection of PCV2 antigen was 8 times more sensitive than WB (Fig. 6E).

### Specificity of the immunochromatographic strip.

**FIG 7:**
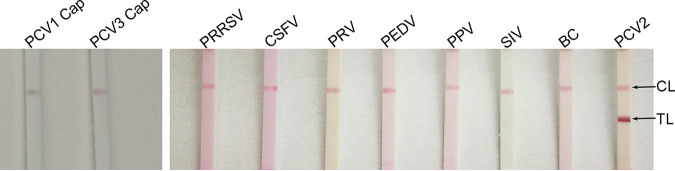
Specificity of the immunochromatographic strip. Uninfected PK15 cells were used as a blank control (BC), and PCV2 was used as a positive control (PC).

The specificity of the strip was determined by testing its cross-reactivity with several other swine viruses or viral proteins, including PRRSV, CSFV, PRV, PEDV, PPV, swine influenza virus (SIV), PCV1 Cap protein, and PCV3 Cap protein. As shown in Fig. 7, two red bands only appeared in the detection for PCV2, indicating a positive result, while for other swine viruses, as well as for PCV1 and PCV3 Cap proteins, all had only one red band at the control line (CL), indicating a negative result. The above-described results suggested that the strip detected PCV2 with high specificity.

### Repeatability of the immunochromatographic strip.

**FIG 8:**
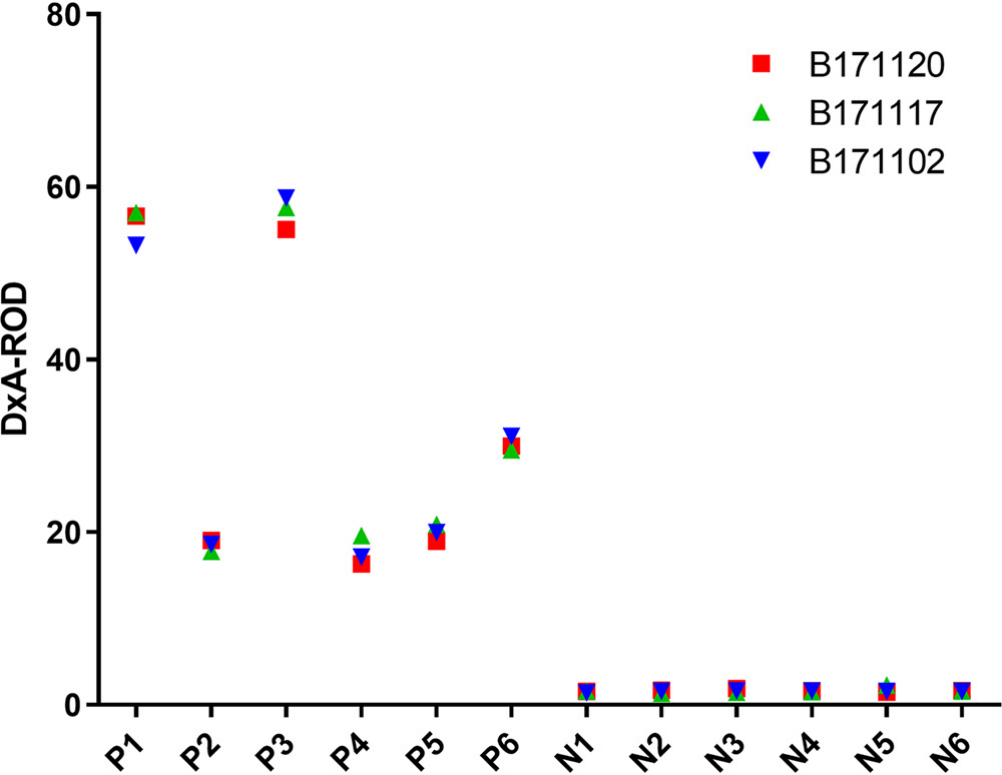
Repeatability of the immunochromatographic strip. P1 to P6, positive samples. N1 to N6, negative samples. B171102, B171117, and B171120 are 3 different batches of the strip.

The repeatability of the strip was evaluated by testing samples in a sample plate using 3 different batches of the strip. As shown in Fig. 8, there was no significant difference in the color intensity of the TL between the three different batches of the strip, indicating that the strip was repeatable.

### Stability of the immunochromatographic strip.

Stored for 3, 6, 9, and 12 months, the strip had the same LOD as the freshly prepared strip when detecting PCV2 strain HN-LB-2016, indicating that the strip was stable (Table 2).

**TABLE 2:** Stability of the immunochromatographic strip for PCV2 antigen detection

Storage time (h)	Sensitivity (PCV2) (lgTCID_50_/mL)	Specificity^*a*^								
		PCV2	PCV1 Cap	PCV3 Cap	PRRSV	CSFV	PRV	PEDV	PPV	SIV
0	3.18	+	–	–	–	–	–	–	–	–
3	3.18	+	–	–	–	–	–	–	–	–
6	3.18	+	–	–	–	–	–	–	–	–
9	3.18	+	–	–	–	–	–	–	–	–
12	3.18	+	–	–	–	–	–	–	–	–

a +, positive result; −, negative result.

## DISCUSSION

In the past nearly 20 years, the widespread application of PCV2 vaccine has effectively improved the production parameters and economic benefits of the vaccinated pigs (19). Several PCV2 vaccines have been marketed, including inactivated vaccines, subunit vaccines, and PCV1-2 chimera vaccines. However, these commercial PCV2 vaccines mainly target a single genotype (20, 21). Currently, the coexistence of PCV2 genotypes such as PCV2a, PCV2b, and PCV2d poses new challenges for the existing PCV2 vaccines (22). PCV2 vaccines have to be updated to ensure their effectiveness. Cap protein-based multivalent or chimeric vaccine is the research direction of the next-generation PCV2 vaccine. The immunochromatographic strip prepared in this study provides a ready to use, labor-saving, and reliable method for real-time monitoring of PCV2 antigen in PCV2 vaccine research.

The immunochromatographic strip is well matched with ASSURED criteria set by the World Health Organization—affordable, sensitive, specific, user-friendly, rapid/robust, equipment-free or minimal, and deliverable to those with the greatest need (23). Owing to its unique advantages, the immunochromatographic strip is an ideal choice for point-of-care tests (POCT), not only for traditional centralized laboratory-based diagnosis, but also for situations where expert staff and special equipment are lacking, providing real-time and on-site detection. AuNPs are the most widely used and well-established markers and have unique properties, including ease of synthesis, high affinity for proteins and biomolecules, good stability, high values for charge transfer, and good optical signal (24). AuNP-based immunochromatographic strips have been widely used as qualitative diagnostic tools for POCT (25). Zhang et al. developed an AuNP-based immunochromatographic strip to detect chicken infectious bursal disease virus in 2 min (26). Li et al. developed an AuNP-based strip for rapid detection of severe acute respiratory syndrome coronavirus 2 spike protein (27). Jin et al. developed a rapid immunochromatographic strip for detection of PCV2 antibodies in 5 min (28). In this study, an AuNP-based immunochromatographic strip for rapid detection of PCV2 antigen (PCV2 virions or Cap protein) was developed for the first time.

Monoclonal antibody is the main component used to determine the performance of AuNP-based immunochromatographic strips. In this study, six MAbs that specifically recognize PCV2 and PCV2 Cap protein were obtained (Fig. 2). The purity of these MAbs obtained by protein G affinity chromatography was all greater than 90%, laying a good foundation for the preparation of PCV2 antigen strip (Fig. 3). A dual-MAb sandwich mode was used to develop the immunochromatographic strip for rapid detection of PCV2 virions or Cap protein with high specificity and no cross-reactivity with other swine viruses (Fig. 4, 6, and 7). The antigen content of commercially inactivated PCV2 vaccines should not be less than 10^5^ TCID_50_/mL, and subunit vaccine should not be less than 100 μg/mL. The LOD of the immunochromatographic strip developed in this study was 10^3,18^ TCID_50_/mL for PCV2 and 2.03 μg/mL for PCV2 Cap protein (Fig. 6B and C). The sensitivity of the immunochromatographic strip was higher than that of WB, which fully met the requirements of PCV2 antigen detection in vaccine research (Fig. 6E). The color shades on the TL reflected antigen content to a certain extent, indicating that the immunochromatographic strip could be used as a semiquantitative detection tool in vaccine research (Fig. 6D). For example, in the process of PCV2 virus reproduction or Cap protein preparation, the developed strip can specifically and rapidly detect the content of PCV2 or PCV2 Cap protein, saving time and money. During the purification process of PCV2 or PCV2 Cap protein, the strip can be used to specifically and rapidly identify whether each wash or elution fraction contains effective antigens, avoiding unnecessary downstream operations. Further work will explore novel labeled nanomaterials to improve the sensitivity of the strip to monitor PCV2 clinical infection.

In conclusion, an AuNP-based immunochromatographic strip for rapid detection of PCV2 antigen (PCV2 virions or Cap protein) was reported for the first time. The strip provided a sensitive, specific, user-friendly, rapid, robust, and equipment-free tool for antigen monitoring in PCV2 vaccine research.

## MATERIALS AND METHODS

### Cells and viruses.

BL21(DE3) competent cells were purchased from TaKaRa Biomedical Technology (Beijing, China). PK-15, human embryonic kidney 293T (HEK293T), Marc145, and MDCK cells were kept in our lab. PCV2 strain HN-LB-2016, PRRSV strain BJ-4, CSFV strain Shimen, PRV strain Tangyin/Henan, PEDV strain CH_hubei_2016, and PPV reference strain 7909 were stored in the Henan Provincial Key Laboratory of Animal Immunology. SIV strain A/swine/Henan/1/2010 was stored in South China Agricultural University. The sources and GenBank accession numbers of these viruses are listed in Table 3.

**TABLE 3:** Virus strains used in this study

Organism	Strain	Collection date	Accession no.
PCV2	HN-LB-2016	2016	MK604485
PRV	Tangyin/Henan	2014	KP009871.1 (gE), KP009883.1 (gC), KP009895.1 (gB)
PRRSV	BJ-4	2000	AF331831.1 (complete genome)
CSFV	Shimen	1999	AF092448.2 (complete genome)
PEDV	CH_hubei_2016	2016	KY928065.1 (complete genome)
SIV	A/swine/Henan/1/2010	2010	KF277766.1 (HA), KF541237.1 (NA)

### Production and characterization of monoclonal antibodies.

Monoclonal antibodies against PCV2 were generated according to the method described previously (29). Briefly, 6- to 8-week-old female BALB/c mice were immunized subcutaneously with 50 μL of a commercial vaccine based on PCV2 Cap protein expressed in a baculovirus expression system at 0, 14, and 28 days post-prime immunization (dpi). Serum samples were collected and measured at 42 dpi by indirect ELISA and IPMA. The mouse with the highest titer was given the same commercial vaccine (100 μL) intravenously. Four days after the last immunization, splenocytes from the mouse were fused with SP2/0 myeloma cells for preparation of hybridoma cells using polyethylene glycol (PEG) 1500. Positive hybridoma cell lines generating the desired antibodies were screened by IPMA and subcloned more than 3 times by the limiting dilution method. The subtypes of these MAbs were detected using the mouse monoclonal antibody subtype identification kit (Proteintech, Wuhan, China). The ability of these MAbs to bind to PCV2 Cap was evaluated by indirect ELISA. Antibody titers of these MAbs were detected by IPMA and the immunochromatographic strip for detection of PCV2 antibodies. The neutralization capacity of these MAbs was assessed by virus neutralization (VN) assay. The cross-reactivity of these MAbs with other porcine viruses, including PRRSV, CSFV, PRV, PEDV, and PPV, was identified by IPMA.

### Selection of the matched antibody pair.

Before being labeled with AuNPs, anti-PCV2 MAbs were purified by protein G affinity chromatography. In order to select the matched antibody pair for the development of the immunochromatographic strip, a dot-strip, mimicking the strip, was used to screen the immobilized antibody and AuNP conjugated antibody. All 6 MAbs were labeled by AuNPs. Unlabeled antibodies act as immobilized antibodies and were dotted on nitrocellulose membrane with 0.3 μL/strip. AuNP-conjugated antibodies were spotted on the conjugate pad with 1 μL/strip. The 2-fold dilution of PCV2 strain HN-LB-2016 (1.95 × 10^5^ TCID_50_/mL) was used to detect the dot strip. The dot strip was placed horizontally for 5 min to observe the result. The color strength of the dots was screened with a TSR-3000 reader (Bio-Dot, California, USA), and the DxA-ROD values were analyzed with AIS software. The antibody pair with the strongest color was selected to prepare the immunochromatographic strip.

### Preparation and characterization of MAb-AuNPs.

AuNPs were prepared using the trisodium citrate method described previously (30). The size and shape of these AuNPs were evaluated by TEM (JEM-1400; Hitachi Ltd., Tokyo, Japan). The MAb-AuNP complex was prepared according to a previously described method (28, 31). First, the pH of the AuNP solution was adjusted to 9.0 by adding 0.2 M K_2_CO_3_. Then, the optimal concentration of anti-PCV2 MAb to stabilize the AuNPs was determined. Briefly, 125 μL of AuNP solution (pH 9.0) was added into each well of the microplate. Subsequently, different dosages of the MAb were added to get concentrations of 80 μg/mL, 40 μg/mL, 20 μg/mL, 10 μg/mL, 5 μg/mL, 2.5 μg/mL, and 1.25 μg/mL. The mixtures were stirred and incubated for 30 min at room temperature. Lastly, 125 μL of 10% (wt/vol) NaCl was added into each well. In this step, the unsaturated AuNP solution flocculated due to the presence of a high salt concentration (32). The optimal MAb concentration for stable AuNPs was determined based on the color variation of the solution. The lowest MAb concentration that did not change the color of the solution after the addition of NaCl was selected as the optimal MAb concentration for AuNP labeling. The MAb at the optimal concentration was then added to the AuNP solution and incubated at room temperature for 30 min to prepare the MAb-AuNP conjugate. Following the conjugation of the MAb with AuNPs, the conjugate was blocked by bovine serum albumin (BSA). Finally, the mixture was centrifuged at 15,000 rpm at 4°C for 30 min. The resulting precipitate was resuspended in 20 mM sodium borate containing 1% (wt/vol) BSA and 0.1% (wt/vol) NaN_3_. The change from AuNPs to MAb-AuNPs was characterized by dynamic light scattering (DLS) (Malvern, Worcestershire, UK) and UV absorption spectra (SpectraMax i3, Molecular Devices, LLC, California, USA).

### Preparation of the immunochromatographic strip.

The strip was mainly composed of four parts: a sample pad, a conjugate pad containing AuNP-labeled MAb, a detection membrane containing a test line (TL) and a control line (CL), and an absorbent pad. The immobilized antibody (0.5 mg/mL) and goat anti-mouse IgG (1 mg/mL) were sprayed onto the preprocessed nitrocellulose membrane at a flow rate of 0.9 μL/cm to prepare the detection membrane. AuNP-labeled-MAb was sprayed onto the preprocessed fiberglass at a flow rate of 5.55 μL/cm to prepare the conjugate pad. Then, the detection membrane was dried at 42°C for 1 h, and the conjugate pad was dried at 42°C for 4 h. The strip was assembled according to previous work (33).

### Detection range and result judgment of the immunochromatographic strip.

The strip can be used to detect PCV2 Cap protein or PCV2 in common buffers or cell culture media. The strip was inserted into the sample solution (100 μL) and placed horizontally for 5 min to observe the result. As shown in Fig. 6A, both the TL and CL turned red, which was judged as positive. If only the CL turned red, the result was judged as negative. No line or only TL turning red indicated that the operation was incorrect or the strip was invalid.

### Sensitivity of the immunochromatographic strip.

The sensitivity of the strip was determined using PCV2 strain HN-LB-2016 and PCV2 Cap protein. PCV2 strain HN-LB-2016 ranging from 10^5.00^ TCID_50_ to 10^2.58^ TCID_50_ and PCV2 Cap protein ranging from 130 μg/mL to 1.02 μg/mL were used to determine the LOD of the strip. The color intensity of the TL was measured with a TSR-3000 reader and was analyzed with AIS software.

### Specificity of the immunochromatographic strip.

To evaluate the specificity of the strip, control experiments were carried out using other swine viruses or viral proteins, including PRRSV, CSFV, PRV, PEDV, PPV, SIV, or PCV1 and PCV3 Cap proteins. PCV2 strain HN-LB-2016 was used as the positive control (PC). Uninfected PK15 cell culture was used as the blank control (BC). All viruses were freeze-thawed three times. After centrifugation, supernatant was taken for detection.

### Repeatability of the immunochromatographic strip.

Repeatability of the strip was evaluated using strips from 3 different batches (lot numbers B171102, B171117, and B171120). The color intensity on the TL was measured with a TSR-3000 reader and analyzed with AIS software. Each sample was tested at least three times with each batch of the strip. The repeatability of the strip was judged according to the test results.

### Stability of the immunochromatographic strip.

The strip was stored in a dry state at room temperature and taken out at 3, 6, 9, and 12 months to assess its stability. PCV2 strain HN-LB-2016 was used to evaluate the sensitivity of the strip. Other swine viruses or viral proteins, including PRRSV, CSFV, PRV, PEDV, PPV, SIV, or PCV1 and PCV3 Cap proteins were used to assess the specificity of the strip.

### Ethics statement.

The animal experiments complied with animal care and ethics guidelines and were authorized and supervised by the Ethical and Animal Welfare Committee of Henan Academy of Agricultural Sciences (approval number SYXK 2021-0003).

### Data availability.

All data presented in this study are available on request from the corresponding authors.
